# Development of the HPLC–ELSD method for the determination of phytochelatins and glutathione in *Perilla frutescens* under cadmium stress conditions

**DOI:** 10.1098/rsos.171659

**Published:** 2018-05-09

**Authors:** Xinyu Zheng, Shen Chen, Meiqin Zheng, Jun Peng, Xiaosan He, Yongming Han, Jingjing Zhu, Qingtie Xiao, Rixin Lv, Ruiyu Lin

**Affiliations:** 1 Fujian Provincial Key Laboratory of Agroecological Processing and Safety Monitoring, School of Life Sciences, Fujian Agriculture and Forestry University, Fuzhou 350002, People's Republic of China; 2 Key Laboratory of Crop Ecology and Molecular Physiology of Fujian Province, Fujian Agriculture and Forestry University, Fuzhou 350002, People's Republic of China

**Keywords:** HPLC–ELSD, glutathione, phytochelatins, *Perilla frutescens*

## Abstract

A rapid, accurate and simple method was developed for the simultaneous determination of glutathione (GSH) and phytochelatins (PCs) by high-performance liquid chromatography (HPLC) with an evaporative light-scattering detector. GSH, phytochelatin 2 (PC_2_), PC_3_, PC_4_, PC_5_ and PC_6_ can be separated with baseline separation within 9 min using a Venusil AA column (250 mm × 4.6 mm i.d., 5 µm particle sizes). Acetonitrile and water containing 0.1% trifluoroacetic acid (0.1%) were employed as the mobile phase for the gradient elution. The drift tube temperature and flow rate of the carrier gas (N_2_) were 50°C and 1.5 l min^−1^, respectively. Under optimum conditions, good linear regression equations of six analytes were obtained with the detection limits ranging from 0.2 to 0.5 µg ml^−1^. The proposed method has been applied successfully for the quantification of GSH and PCs in *Perilla frutescens* (a cadmium hyperaccumulator) under cadmium stress. The recoveries were between 82.9% and 115.3%.

## 1. Introduction

Phytoremediation is a technology of phytoextraction, which was developed recently for removing excessive heavy metal ions from contaminated environments using a hyperaccumulator [1–3]. It has become a hot topic in current research. As is well known, cadmium is one of the most toxic heavy metals. Exposure to cadmium can cause serious damage to several organs, such as kidneys, liver and lungs [4]. In recent years, much attention has been paid to the more severe problem of cadmium-polluted soil in the People's Republic of China, so phytoremediation of cadmium-polluted soil by using a hyperaccumulator presents a promising alternative technology to current environmental governance. *Perilla frutescens* (L.) Britt. (Labiatae, Herb) had been proved to be a cadmium hyperaccumulator in our previous studies. The enrichment coefficient of *Perilla frutescens* was greater than 50. The protective enzyme system of *Perilla frutescens* responded positively to cadmium stress, but the biomass was obviously inhibited [5,6]. Phytochelatins (PCs) are a class of cysteine-rich low-molecular-weight polypeptides that are synthesized enzymatically from glutathione (GSH) and play an important role in the response to excess levels of heavy metal ions in the cytoplasm of plants. The PCs, whose general formula is (γ-Glu-Cys)_*n*_-Gly (the value of *n* ranging from 2 to 11), can form complexes with heavy metals ions such as Cd^2+^, Pb^2+^, Zn^2+^, Ag^+^ or Hg^2+^. The molecular weight of the as-formed complexes is between 2500 and 3600 [7]. The detoxification mechanism in the present work has been illustrated by several previously reported works [8–10]. For example, when plant cells and tissues are exposed to heavy metal ions, PCs can be rapidly synthesized by the induction of heavy metal ions. Afterwards, PCs and heavy metal ions can combine together to form non-toxic compounds, which are then moved into the vacuole by active transport. After the degradation of PCs, the heavy metal ions are retained in the vacuole, from which the plant cells can be protected [11]. So, the concentration determination of PCs in *Perilla frutescens* is of great practical importance to reveal the mechanism of Cd(II) tolerance and detoxification in *Perilla frutescens*. The most common method described for the determination of PCs is using high-performance liquid chromatography (HPLC) combined with several detectors, such as ultraviolet–visible spectrum [12,13], fluorescence [14–16], electrochemistry [17–19] or mass spectrometry (MS) [20,21]. However, a proper derivatization procedure, which is necessary to provide a specific chromogenic or fluorogenic compound, is needed for the determination of thiol compounds using an ultraviolet–visible or fluorescence detector. The electrodes often need to be modified to improve the sensitivity and reproducibility with electrochemical detection. The MS method is the most effective, but this detector is quite expensive. The evaporative light-scattering detector (ELSD) is a universal mass detector that has been widely applied in many fields, such as food analysis, pharmaceutical analysis and environmental analysis [22–24]. ELSD can be applied to quantitatively detect solutes which should have a higher volatility than the mobile phase via the detection of light scattering changes. The response values are only associated with the mass of the solutes. ELSD is attractive as it does not involve a derivatization procedure for online quantification of biological samples. So far, there have been no reports as far as we are aware that simultaneously analyse PCs and GSH in plants using HPLC coupled with an ELSD. In this paper, a rapid, accurate and simple HPLC–ELSD method was developed for the determination of GSH and PCs in *Perilla frutescens* under cadmium stress. The establishment of this method can provide a good reference to study the hyperaccumulative characteristics of this hyperaccumulator.

## 2. Experimental

### 2.1. Reagents

GSH and dithiothreitol (DTT) were obtained from the Chinese Institute of Biological Products Control (Beijing, China). Phytochelatin 2 (PC_2_), phytochelatin 3 (PC_3_), phytochelatin 4 (PC_4_), phytochelatin 5 (PC_5_) and phytochelatin 6 (PC_6_), purity 95%, were purchased from AnaSpec Inc. (Fremont, CA, USA). Other reagents were of analytical grade.

### 2.2. Instrumentation

The HPLC instrument used in this experiment was an Agilent 1260 series unit consisting of a vacuum degasser, quaternary pump, auto sampler and column compartment with oven (Agilent Technologies, Santa Clara, CA, USA). The HPLC detector was an Alltech 3300 ELSD (Alltech Associates, USA). A Venusil AA column (250 mm × 4.6 mm i.d., 5 µm particle size; Tianjin Bonna-Agela Technologies Co. Ltd.) was used for the separation.

### 2.3. Chromatographic conditions

The mobile phase consisted of acetonitrile (A) and water containing 0.1% trifluoroacetic acid (0.1% TFA, B). A gradient elution process was employed to separate the PCs and GSH. The gradient elution process included 10%A to 30%A (0–10 min), 30%A to 100%A (10–15 min), 100%A to 10%A (15–20 min), 10%A (20–25 min) at a flow rate of 0.8 ml min^−1^. The injection volume, column temperature, temperature in the drift tube and flow rate of the carrier gas were 10 µl, 30°C, 50°C and 1.5 l min^−1^, respectively.

### 2.4. Plant material and sample preparation for HPLC

Seeds of *Perilla frutescens* and rice were first germinated on the experimental soil in a greenhouse. After 3 days of germination, the uniform seedlings of *Perilla frutescens* were transplanted into a plastic basin filled with 10 l Hoagland hydroponic solution (served as the normal nutrient condition). When the seedling reached 10 cm in length, Cd(II) (5 mg l^−1^) was added to the nutrient solution. Stems of *Perilla frutescens* and roots of rice were collected after three weeks under cadmium stress. The stem of *Perilla frutescens* and the roots of rice were selected and cleaned carefully with 0.1 mol l^−1^ EDTA solution and then the fresh weight was obtained. The plant tissue was frozen in liquid nitrogen immediately in order to disrupt the cell walls. The samples were stored in a freezer at −80°C.

The samples (approximately 0.2 g) were ground with liquid nitrogen. Subsequently, 1.8 ml of 0.1% TFA and 0.2 ml of 200 mmol l^−1^ DTT were added to the samples to extract the GSH and PCs. The homogenate was centrifuged at 12 000 r.p.m. for 10 min at 4°C. The supernatant was filtered through a 0.22 µm filter membrane. The filtrate was injected into the Venusil AA column for the HPLC–ELSD analysis.

## 3. Results and discussion

### 3.1. Method optimization

The most common mobile phase used to separate the PCs is a mixture of A with 0.1% TFA [25–27], so A and 0.1% TFA were selected for the mobile phase. Different proportions of A and 0.1% TFA were investigated to separate the six analytes. The results showed that the retention time was shorter with an increasing percentage of acetonitrile. The best separation was found when the proportion of A to 0.1% TFA was 10 : 90 (V : V). However, the analysis time was more than 20 min. The gradient elution technique possesses some merits in the separation of mixed organic compounds. The peaks whose retention time was short appeared later via an eluent of low organic phase--high water phase so as to separate the mixed organic compounds, while the peaks with a long retention time long appeared in advance via an eluent of high organic phase–low water phase. The use of this technique not only saved time, but also made the peaks narrower, thus making the integration of the peaks more accurate. Therefore, a general gradient elution process was used. The gradient profile was the following: 10%A to 30%A (0–10 min), 30%A to 100%A (10–15 min), 100%A to 10%A (15–20 min), 10%A (20–25 min) at a flow rate of 0.8 ml min^−1^. Under these gradient elution conditions, the longest analysis time was 9 min.

**Figure 1.**
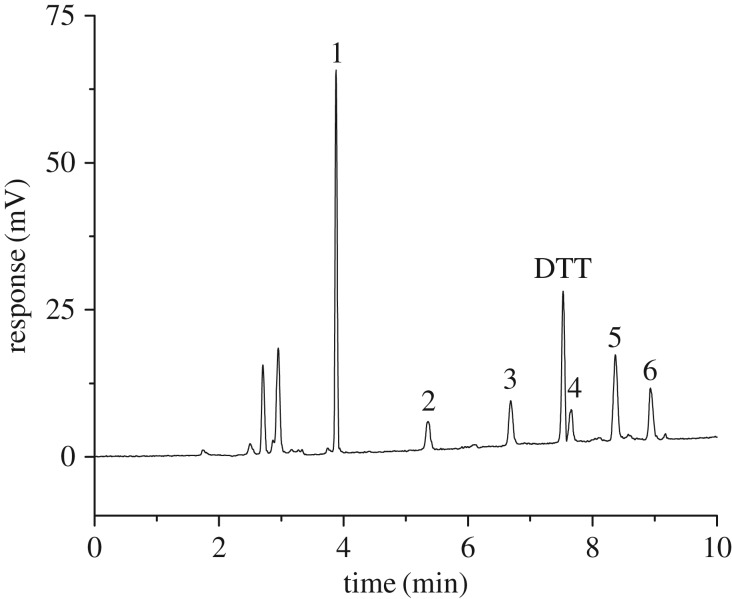
HPLC–ELSD chromatogram of a standard mixed solution. 1—GSH (20 µg ml^−1^), 2—PC_2_ (20 µg ml^−1^), 3—PC_3_ (20 µg ml^−1^), 4—PC_4_ (20 µg ml^−1^), 5—PC_5_ (20 µg ml^−1^), 6—PC_6_ (20 µg ml^−1^). The gradient elution process was: 10%A to 30%A (0–10 min), 30%A to 100%A (10–15 min), 100%A to 10%A (15–20 min), 10%A (20–25 min) at a flow rate of 0.8 ml min^−1^; column temperature: 30°C; temperature in the drift tube: 50°C; flow rate of the carrier gas: 1.5 l min^−1^; sample injection volume: 10 µl.

The flow rate of nebulizer gas and the temperature of the drift tube are two other important parameters which could greatly influence the sensitivity and reproductivity of ELSD. The droplets are transported from the atomization chamber to the drift tube by a carrier gas for evaporation. In the drift tube, the solvent was completely removed, leaving the particulate or pure solutes. The flow rate of the carrier gas affects the formation of droplets in the atomizer, while the temperature in the drift tube determines whether the evaporation of the mobile phase was complete, both of which affect the response of the detector. Flow rates of the nebulizer gas (1.0, 1.2, 1.4, 1.5, 1.6, 1.8 and 2.0 l min^−1^) and the temperature of the drift tube (40, 45, 50, 55, 60°C) were studied. When the carrier gas pressure is low, spikes in the chromatographic peaks are generated because of the unevenly sprayed droplets. The greater the flow rate of the carrier gas, the smaller the size of droplets, thus the sensitivity of the ELSD would decrease. Because the temperature in the drift tube was too low, leading to incomplete evaporation of the mobile phase, part of the mobile phase flowed out directly from the evaporation tube. This results in a weaker detection signal. If the temperature is too high, the size of the particles is uneven, resulting in poor precision. Good peak symmetries and high sensitivity can be obtained when the flow rate of the nebulized gas is 1.5 l min^−1^ and the temperature of the drift tube is 50°C. In addition, setting the gain value to 1 could increase the signal response and the baseline noise does not have any effect on the detection. A standard chromatogram (figure 1) of PCs and GSH was achieved under the optimized conditions.

### 3.2. Calibration curves, linear ranges and detection limits

Different mass concentrations of a mixed standard solution were determined by HPLC–ELSD for the construction of calibration curves and linear ranges under the optimum conditions. The concentrations of six analytes were proportional to the peak areas. The calibration parameters for the simultaneous determination of six analytes are listed in table 1. As seen in table 1, all the calibrations showed good linearity (*r*^2^ ≥ 0.9991) and all the linear ranges had two orders of magnitude and the detection limits ranged from 0.2 to 0.5 µg ml^−1^. These results suggested that the developed method for the determination of the six analytes was accurate and sensitive. Compared with other reported methods [27–31], similar response ranges but higher detection limits were achieved with the HPLC–ELSD method compared with the HPLC–fluorescence (Flu) method. However, the HPLC–Flu method has a complicated derivatization process, whereas low detection limits can be obtained with the HPLC–ELSD method relative to the HPLC–ED or ion-pair chromatography (IPC)–MS methods (table 2).

**Table 1.** Calibration curves, linear ranges and detection limits.^a^ Chromatographic conditions are the same as in figure 1.

compounds	regression equation	correlation coefficient (*r*^2^)	linear range (µg ml^−1^)	detection limits (µg ml^−1^)
GSH	*y* = 7.565*x* − 2.2469	0.9994	1–100	0.2
PC_2_	*y* = 1.5084*x* − 0.5623	0.9993	2–100	0.5
PC_3_	*y* = 2.082*x* − 0.621	0.9999	2–100	0.5
PC_4_	*y* = 1.0385*x* − 0.3817	0.9991	2–100	0.5
PC_5_	*y* = 4.7937*x* − 0.2713	0.9999	2–100	0.5
PC_6_	*y* = 2.712*x* − 1.2568	0.9999	2–100	0.5

^a^*y*-axis represents the value of the peak area, and the *x*-axis expresses the value of the concentration.

**Table 2.** Comparison of the reported methods in the literature. AD, amperometric detection; ED, electrochemical detection.

	linear range (µg ml^−1^)						detection limits (µg ml^−1^)						
method	GSH	PC_2_	PC_3_	PC_4_	PC_5_	PC_6_	GSH	PC_2_	PC_3_	PC_4_	PC_5_	PC_6_	reference
HPLC–ED							1.13	1.73	2.88	2.23	2.08		[25]
HPLC–ED							0.83	1.02	3.16	3.46	6.06		[26]
ICP–MS								0.54	0.78	0.51	1.13	5.67	[27]
HPLC–AD							0.35	0.63	2.15				[28]
HPLC–Flu	0.03–3	0.05–0.5	0.08–0.8	0.1–1			0.02	0.03	0.03	0.03			[29]
HPLC–ELSD	1–100	2–100	2–100	2–100	2–100	2–100	0.2	0.5	0.5	0.5	0.5	0.5	this work

### 3.3. Reproducibility and stability

Three consecutive injections of six analytes were performed to evaluate the reproducibility of the developed method. The experimental results showed that the relative standard deviations (RSDs) of the signal response and migration time were less than 1.5% and 0.4% (table 3), suggesting that the established method exhibits good reproducibility. The determinations at given times (0, 24, 48, 72, 96 and 120 h) were used to assess the stability of the sample solutions (table 4). The results demonstrated that the signals of the six analytes were all stable but declined a little within 5 days due to the addition of antioxidant (DDT).

**Table 3.** Inter-day (*n* = 3) precision for six analytes with the same standard solution.^a^ Chromatographic conditions are the same as in figure 1.

	peak area					retention time (min)				
compound	1	2	3	mean	RSD (%)	1	2	3	mean	RSD (%)
GSH	149.6	148.7	149.1	149.1	0.3	3.879	3.871	3.875	3.875	0.4
PC_2_	29.4	29.4	29.9	29.6	1.0	5.359	5.354	5.355	5.356	0.3
PC_3_	41.6	40.5	40.9	41.0	1.4	6.686	6.686	6.688	6.687	0.1
PC_4_	20.7	20.1	20.5	20.4	1.5	7.659	7.650	7.655	7.655	0.4
PC_5_	94.9	95.8	96.1	95.6	0.7	8.367	8.369	8.371	8.369	0.2
PC_6_	53.5	52.9	52.7	53.0	0.8	8.930	8.933	8.931	8.931	0.2

^a^The concentration of GSH, PC_2_, PC_3_, PC_4_, PC_5_, PC_6_ and PC_6_ was 20 µg ml^−1^.

**Table 4.** The stability of six standard analytes at given times (*n* = 3).^a^ Chromatographic conditions are the same as in figure 1.

	GSH	PC_2_	PC_3_	PC_4_	PC_5_	PC_6_
0 h	151.7	30.4	42.9	20.7	94.5	53.1
24 h	149.2	28.9	34.7	19.9	96.4	51.6
48 h	148.2	28.4	29.5	19.7	89.7	48.6
72 h	147.4	27.5	28.9	19.6	86.4	46.7
96 h	143.2	26.3	26.2	19.4	83.7	45.2
120 h	141.3	25.3	25.7	18.7	82.5	43.6

^a^The concentration of GSH, PC_2_, PC_3_, PC_4_, PC_5_, PC_6_ and PC_6_ was 20 µg ml^−1^.

### 3.4. Real sample analysis

**Figure 2.**
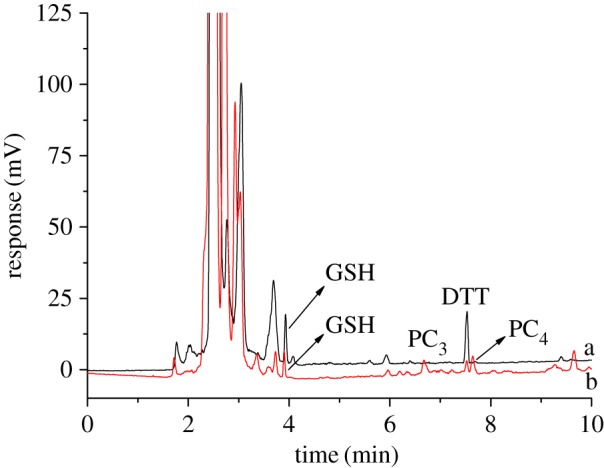
Chromatogram for the stems of *Perilla frutescens*. a—non-stress; b—cadmium stress. Chromatographic conditions are the same as in figure 1.

**Figure 3.**
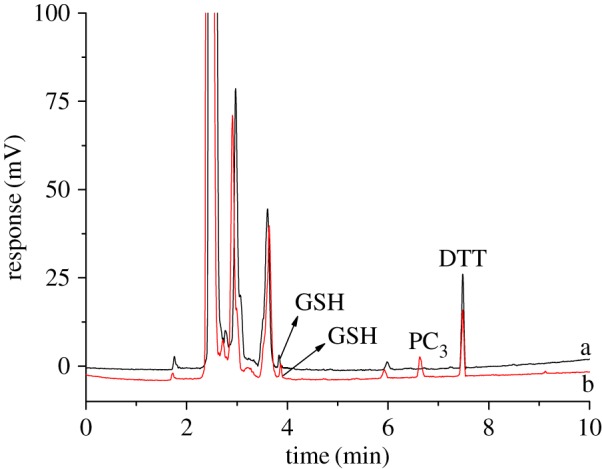
Chromatogram for the roots of rice. a—non-stress; b—cadmium stress. Chromatographic conditions are the same as in figure 1.

The stems of *Perilla frutescens* and the roots of rice under cadmium stress and non-stress were treated as described in §2.4. Real samples were analysed by HPLC–ELSD under the optimum conditions. As shown in figure 2, GSH, PC_3_ and PC_4_ were detected in the stem of *Perilla frutescens* under cadmium stress. Correspondingly, only GSH could be found in the stem of *Perilla frutescens* under non-stress. To further confirm the universality for the developed method, GSH and PCs in the roots of rice under cadmium stress and non-stress were also determined by HPLC–ELSD. As seen in figure 3, GSH and PC_3_ can be found in the roots of rice under cadmium stress conditions, while only GSH was determined in the roots of rice. The contents of GSH and PCs are listed in table 5. To evaluate the accuracy of the developed method, the recoveries were determined by the addition of standard stock solution to the real samples of *Perilla frutescens* under cadmium stress. The recovery can be calculated as

$$\text{recovery}\;(\%)=\frac{\text{response of analyte spiked into matrix (processed) }}{\text{response of pure standard analyte (unprocessed) }}\times 100.$$

**Table 5.** Contents of GSH and PCs in the stems of *Perilla frutescens* and roots of rice (*n* = 3).^a^ 1—stems of *Perilla frutescens* under non-stress; 2—stems of *Perilla frutescens* under cadmium stress; 3—roots of rice under non-stress; 4—roots of rice under cadmium stress.

	GSH		PC_2_		PC_3_		PC_4_		PC_5_		PC_6_	
samples	µg ml^−1^	µg g^−1^	µg ml^−1^	µg g^−1^	µg ml^−1^	µg g^−1^	µg ml^−1^	µg g^−1^	µg ml^−1^	µg g^−1^	µg ml^−1^	µg g^−1^
1	4.67 ± 0.15	46.7 ± 1.52	—	—	—	—	—	—	—	—	—	—
2	3.10 ± 0.11	31.0 ± 1.14	—	—	11.30 ± 0.50	113.0 ± 5.11	—	—	23.48 ± 0.77	234.8 ± 7.70	—	—
3	1.40 ± 0.07	14.0 ± 0.70	—	—	—	—	—	—	—	—	—	—
4	1.51 ± 0.06	15.1 ± 0.63	—	—	12.64 ± 0.65	126.4 ± 6.54	—	—	—	—	—	—

^a^Chromatographic conditions are the same as in figure 1.

The experimental data suggested that the average recoveries of six analytes were in the range of 82.9–115.3% and the RSD was lower than 3.7% (table 6). The analytical results were satisfactory.

**Table 6.** Recovery of six analytes in the stem of *Perilla frutescens* (*n* = 3).^a^

compound	content in *Perilla* stem (µg ml^−1^)	added (µg ml^−1^)	found (µg ml^−1^)	recovery (%)	RSD (%)
GSH	3.10 ± 0.11	2.0 ± 0.06	5.88 ± 0.18	115.3	3.2
		20.0 ± 0.54	19.16 ± 0.68	82.9	3.1
PC_2_	0	2.0 ± 0.07	2.11 ± 0.07	105.5	3.5
		20.0 ± 0.64	19.84 ± 0.58	99.2	3.2
PC_3_	11.30 ± 0.42	2.0 ± 0.06	14.55 ± 0.36	109.4	3.7
		20.0 ± 0.55	28.41 ± 0.71	90.8	2.1
PC_4_	23.48 ± 0.77	2.0 ± 0.06	23.6 ± 0.94	92.6	3.3
		20.0 ± 0.59	40.77 ± 1.28	93.8	2.8
PC_5_	0	2.0 ± 0.06	1.78 ± 0.06	89.0	2.9
		20.0 ± 0.42	18.54 ± 0.52	92.7	1.6
PC_6_	0	2.0 ± 0.05	2.14 ± 0.04	107.0	3.1
		20.0 ± 0.87	19.63 ± 0.88	98.2	3.4

^a^Chromatographic conditions are the same as in figure 1.

The real sample tests showed that the established method for the simultaneous determination of GSH and PCs was reliable.

## 4. Conclusion

A simple and rapid method based on HPLC–ELSD was established for the simultaneous determination of GSH and PCs. The method did not need complicated sample preparation. The accuracy and precision of the developed method seem satisfactory. The method cannot be used for monitoring the dynamics of GSH and PCs in *Perilla frutescens* under cadmium stress, but provides technical support for further illuminating the super-accumulation effect of cadmium ions in *Perilla frutescens*.

## Data accessibility

Our data are deposited in the Dryad Digital Repository (https://doi.org/10.5061/dryad.h8b3c) [32].
